# Self‐administration of injectable contraceptives: a systematic review

**DOI:** 10.1111/1471-0528.14248

**Published:** 2016-08-23

**Authors:** CR Kim, MS Fønhus, B Ganatra

**Affiliations:** ^1^ Department of Reproductive Health and ResearchWorld Health OrganizationGenevaSwitzerland; ^2^ Knowledge Centre for the Health ServicesNorwegian Institute of Public HealthOsloNorway

**Keywords:** Contraception, injectable, self‐administration

## Abstract

**Background:**

The contraceptive injectable is a safe and effective method that is used worldwide. With the variety of injectable delivery systems, there is potential for administration by the woman herself. Self‐administration of the contraceptive injectable is the subject of this systematic review.

**Objectives:**

To assess how effective and safe the contraceptive injectable method is when women themselves perform/administer it, compared with when the usual healthcare providers administer it.

**Search strategy:**

We searched PubMed, Popline, Cochrane, CINAHL, and Embase for articles with subject headings or text words related to ‘self‐administration’ and ‘contraception’.

**Selection criteria:**

Studies that compared the administration of the contraceptive injectable by the woman herself versus administration by the healthcare provider were included. Outcomes of interest were continuation rates, safety, and the women's overall satisfaction with the contraceptive provider and method.

**Data collection and analysis:**

We undertook data extraction, descriptive analysis, and assessment of risk of bias.

**Main results:**

Three studies met the inclusion criteria. The best available evidence shows that there may be little or no difference in continuation rates when women self‐administer contraceptive injections (326 per 1000 women; 95% CI 192–554 per 1000 women) compared with administration by healthcare providers (304 per 1000 women). Safety was not estimable as no serious adverse events were reported in any of the studies. With regards to overall satisfaction towards the provider and the method, the effect of the intervention was uncertain.

**Authors’ conclusions:**

Findings suggest that with appropriate information and training the provision of contraceptive injectables for the woman to self‐administer at home can be an option in some contexts.

**Tweetable abstract:**

This review assessed the continuation rates and safety of self‐administration of the contraceptive injection.

## Tweetable abstract

This review assessed the continuation rates and safety of self‐administration of the contraceptive injection.

## Introduction

Contraceptive injectables, both combined hormonal and progestogen only, offer safe and effective reversible contraception.^1^ Over 40 million women worldwide use contraceptive injectables, and in many low‐resource countries, injectables account for at least one‐half of modern use.^1^, ^2^ Combined hormonal contraceptive injectables containing medroxyprogesterone and estradiol cyprionate require intramuscular administration once monthly, and use is most prevalent throughout Latin America.^3^ In Eastern and Southern Africa, progestogen‐only injectables (POIs) account for up to 40% of contraceptive use.^2^ Currently, there are two formulations of POIs referenced in WHO recommendations: depot medroxyprogesterone (DMPA) and norethisterone enantate (NET‐EN).^1^, ^4^ The most widely available POI worldwide is DMPA, which can be administered either intramuscularly (IM) or subcutaneously (SC) every 3 months for contraceptive protection. These formulations are distinct, but therapeutically equivalent.^5^, ^6^ NET‐EN requires intramuscular injection every 2 months.^7^

Features like effectiveness, reversibility, relatively long‐acting effects, and discrete administration contribute to their popularity; however, many current and potential users of injectable contraceptives confront numerous barriers to accessing these methods, particularly in developing regions.^7^ Traditionally, contraceptive injectables have been provided in clinical settings by trained healthcare personnel. Innovations to the injectable delivery system, as well as the availability of a newer subcutaneous formulation of DMPA, have simplified the process, making it possible to consider the engagement of a wider range of healthcare providers, and even women themselves, in the provision of these methods outside of a clinical setting.^8^

Self‐administration of the contraceptive injectable has the potential to positively affect the uptake of a contraceptive. The potential benefit of self‐administration of the contraceptive injectable is increased compliance, by removing the barrier of the woman going to the clinic to receive the injection. In turn, this can increase the likelihood of receiving the injection in a timely fashion and maintaining contraceptive usage.^9^ In addition, self‐administration appears to be an acceptable concept amongst contraceptive users. In a questionnaire survey of women currently using DMPA, a high percentage of women noted a preference for the self‐administration option.^10^

Given the public health importance of this topic, we undertook a systematic review to compare the continuation rates, safety, and satisfaction associated with the self‐administration of contraceptive injectables compared with administration by clinic‐based healthcare providers.

## Methods

We searched PubMed, Popline, Cochrane, CINAHL, and Embase databases, from inception to 22 October 2015, for articles in any language with subject headings or text words related to ‘self‐administration’ and ‘contraception’ (Appendix S1). Randomised controlled trials, comparative observational studies (cohort and case–control), and controlled before‐and‐after studies including women of reproductive age choosing to initiate or continue with the contraceptive injectable, and that reported continuation rates, safety, or satisfaction associated with self‐administration compared with administration of a contraceptive injectable by a healthcare provider, were eligible for inclusion.

We included outcomes comparing the effectiveness of the intervention (self‐administration versus healthcare provider) by looking at continuation rates of the injectable. We also included the incidence of pregnancy arising from a failure in the method as part of our reporting of serious adverse events, in order to assess clinically significant dosing errors from incorrect injection. Results pertaining to the reported mechanics of injection or serum hormone levels following injection were included as indirect evidence for both of these outcomes. We defined serious adverse events as any resulting condition requiring further medical treatment or hospitalisation. We also included reports of minor complications, such as anxiety, pain, and injection‐site reactions, which are known to be more prevalent among women exposed to subcutaneous versus intramuscular injections.^6^, ^11^ We documented self‐reports of satisfaction with the experience of self‐administration compared with administration by a health provider, but we also included reports of overall satisfaction with the method or service when noted.

We reviewed titles and abstracts and the full article, when necessary, to identify studies for inclusion. We hand‐searched reference lists from identified articles and key review articles. We abstracted data from the selected studies using a standardised form.

We determined risk of bias in individual studies based on the criteria outlined in the *Cochrane Handbook for Systematic Reviews of Interventions*.^12^ Two authors independently assessed the overall quality and certainty of the evidence by using the Grading of Recommendations‐Assessment, Development and Evaluation (GRADE) system. This approach takes into account five aspects (study limitations, consistency of effect, imprecision, indirectness, and publication bias) to determine the quality of the body of evidence for each outcome. Evidence was downgraded from ‘high quality’ by one level for serious, or by two levels for very serious, limitations, depending on assessments of the aforementioned aspects. The GRADE profiler (grade 2014) was used to import data from review manager 5.3 (revman 2014) to create GRADE evidence profiles and simplified summary‐of‐findings tables.

## Results

**Figure 1:**
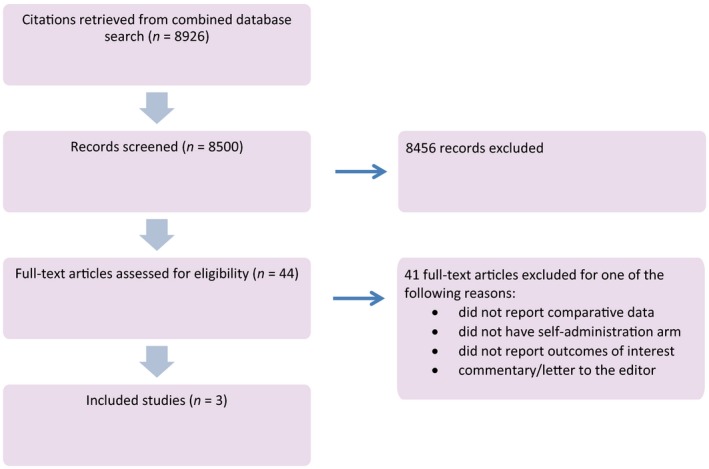
Flow diagram.

We identified a total of 8926 unique citations, and of those citations, 44 underwent full‐text review. Three studies, one randomised controlled trial (RCT) and two prospective cohort studies, met the inclusion criteria for this review (Figure 1).^13^, ^14^, ^15^ Two studies evaluated the self‐administration of DMPA SC, compared with DMPA SC or DMPA IM administered by a healthcare provider; the third study included women self‐administering versus nurse administration of a combined hormonal contraceptive IM injectable.^15^ All three studies took place in high‐resource urban settings.

Beasley et al. randomised eligible participants (*n* = 132) to either self‐administration (*n* = 86) or healthcare provider (*n* = 46) for injection of DMPA SC every 3 months (Table 1). To be eligible, participants needed to express an interest in using DMPA, and could be current, past, or new users of the method. A baseline questionnaire collecting data about demographic characteristics, reproductive characteristics, contraceptive history, and future plans for pregnancy was completed on the day of enrolment, and all participants received a DMPA injection as timed for continuation or according to a conventional start or Quickstart protocol.^16^ Those randomised to self‐administration received verbal and written instructions for self‐injection in the abdomen or anterior thigh from the study coordinator, and then performed the initial injection under supervision. If performed correctly, the woman was given supplies (pre‐packaged subcutaneous DMPA, alcohol pads, bandage, urine pregnancy test, and sharps disposal container, with instructions on safe needle disposal) and a DMPA calendar with dates for the next injection at home. Women in the healthcare provider group received an appointment for their next injection at the clinic. All participants received appointments at 6 and 12 months. The 6‐month visit provided an opportunity to re‐evaluate proficiency with injection in the self‐administration group; if deemed satisfactory, they were given additional supplies for home administration of the next injection. For the healthcare provider group, chart reviews were performed to verify DMPA administration. At both the 6‐ and 12‐month visits, blood was collected to measure serum medroxyprogesterone acetate (MPA) levels.

**Table 1:** Characteristics of included studies

Study design	Country, study period	Length of follow‐up	Intervention and participants	Risk of bias assessment
Randomised controlled trial^13^	USA, 2010–2011	12 months follow‐up with all participants having follow‐up visits at 6 and 12 months	Women were randomised in a 2 : 1 ratio to self‐administration or healthcare provider‐administered DMPA SC, injections were once every 3 months Healthcare provider group: 46 women Self‐administration group: 86 women	Not serious
Pilot cohort study^14^	Scotland, 2008–2010	In the self‐administration group outcomes were measured with monthly phone follow‐up; in the healthcare provider group outcomes were measured every 3 months at the clinic visit Questionnaire at 12 months for both groups	Women were existing DMPA users who expressed interest in self‐injection Injections were every 3 months Healthcare provider group: 64 women Self‐administration group: 58 women	Serious
Prospective cohort study with crossover^15^	USA, 2002–2004	Clinic visit after three cycles of self‐administration, then two more clinic visits for the nurse‐administered doses	The same group of women performed self‐administration for the first 3 months, then the nurse administered the injection for the following 3 months An intramuscular formulation containing DMPA and estradiol cypionate was administered monthly. Healthcare provider group: 10 women Self‐administration group: same 10 women	Serious

A total of 115 women completed follow‐up (87%); ten women in the self‐administration group and six in the healthcare provider group were lost to follow‐up. The majority of women were using DMPA at the end of the study (self, 71%; provider, 63%, *P* = 0.47). The investigators noted that only 47 and 49% of women reported continuous, uninterrupted use at 1 year in the self‐administration and healthcare provider groups, respectively. No method failures, serious adverse events, or minor complications with injection were mentioned, and average serum trough levels of DMPA were comparable between both groups of continuous, uninterrupted users. Satisfaction outcomes were not explicitly reported; however, the authors did report on women switching or discontinuing, which can be treated as an indication of dissatisfaction with the method. Some women in both the self‐administration (*n* = 3) and healthcare provider (*n* = 4) arms switched from SC to IM DMPA administered in a clinic setting. Two of the women in the self‐administration group who crossed over to DMPA IM expressed dislike or discomfort, particularly with self‐injection. The women not using DMPA at the end of the study either switched to another method or discontinued the method because they were seeking pregnancy or did not feel that they were at risk (Table 2).

**Table 2:** Main outcome findings of included studies

Study	Mode of outcome measurement	Findings			
**Continuation rates**					
Beasley et al.^13^	Chart review and clinic follow‐up at 6 and 12 months	**Outcome**	**Self/home (** ***n*** ** = 86)**	**HCP/clinic (** ***n*** ** = 46)**	***P***
		DMPA use at 1 year	61 (71%)	29 (63%)	0.47
		Uninterrupted DMPA use at 12 months	28/61 (47%)	14/29 (48%)	0.70
		MPA* level	686.2	695	0.85
Cameron et al.^14^	Phone follow‐up 2 weeks after injection date for the self‐administration group Questionnaire at end of study for both groups (12 months)	**Outcome**	**Self/home (** ***n*** ** = 58)**	**Clinic (** ***n*** ** = 64)**	***P***
		Discontinuation rate at 12 months	7 (12%)	14 (22%)	0.23
		Self‐injections were given within appropriate intervals			
Stanwood et al.^15^	Study calendar for the three self‐injection months, phone follow‐up on cycle day 30, two surveys (once after self‐injection period, then after clinic visit period)	All subjects complied with dosing regimen schedule with no late or missed injections during the home or clinic phases			
**Safety**					
Beasley et al.^13^		**Severe adverse events:** Not reported **Method failure (e.g. pregnancy):** Not reported			
Cameron et al.^14^	Injection problems reported by the self‐administration group	**Injection problem for self‐administration group**		**Number of women (%)**	
		Injection system (needle detachment or resistance when pushing syringe)		13 (20%)	
		Injection site acute reaction		4 (6%)	
		Injection site skin changes		6 (9%)	
		Self‐administration group had five women withdrawn for mild adverse events **Severe adverse events:** Not reported **Method failure (e.g. pregnancy):** Not reported			
Stanwood et al.^15^	Reporting of pain and worry	Similar low pain and worry with self‐injection and with nurse administration **Severe adverse events:** Not reported **Method failure (e.g. pregnancy):** Not reported			
**Satisfaction**					
Beasley et al.^13^	Exit interview to assess satisfaction at 12‐month visit	Two women in self‐administration group expressed dislike with self‐injection No other results reported from satisfaction survey questions			
Cameron et al.^14^	Questionnaire assessing satisfaction at end of study (12 months or earlier if patient exited study earlier)	**Reported satisfaction**		**Self/SC group**	**HCP/IM group**
		I feel same or better		95%	98%
		I am extremely or somewhat satisfied		93%	97%
		I would recommend this to a friend		95%	100%
		I want to continue this method		90%	91%
Stanwood et al.^15^	Survey after home self‐administration period; additional survey after nurse‐administered phase	Subjects were equally satisfied with the home injections and office injections After home phase, all subjects preferred self‐injection at home to nurse administration After home phase, 8/10 still preferred self‐injection 9/10 women would recommend self‐administration to other women			

HCP, healthcare provider; IM, intramuscular; MPA, medroxyprogesterone; SC, subcutaneously.

Serum trough level of medroxyprogesterone levels in DMPA‐SC continuers at 12 months (measured in pg/mL).

The second study was a controlled cohort pilot study conducted in Scotland.^14^ The women were existing intramuscular DMPA users who expressed an interest in self‐injection (Table 1). A total of 128 women agreed to participate: 64 performed self‐administration and 64 continued with administration by the healthcare provider in a healthcare setting. Women in both groups had similar demographic characteristics and had similar duration of use of DMPA IM at the time of recruitment. The self‐administration group used the subcutaneous formulation of DMPA and the healthcare provider group continued the intramuscular formulation (DMPA IM). The women in the self‐administration group received instructions from the study research nurse using a teaching model that consisted of a belt with artificial skin that could be worn on different parts of the body. When women were seen as competent in the technique on the model, they performed the first injection under supervision at the healthcare setting. They were then given three prefilled syringes of DMPA SC with a list of dates for when the next three injections would be due. They also received information on the self‐injection method and a disposal container for the needle. All women in the self‐administration group received a text message 1 week prior to their scheduled date of injection as a reminder. A study nurse then contacted them by telephone 2 weeks after the date of their scheduled self‐administered injection. This allowed an opportunity to ask whether the woman had self‐injected the DMPA SC, the date of the injection, and if they had experienced any problems.

The main outcome was the discontinuation rate of the method at 12 months. Satisfaction was also recorded, with a questionnaire distributed at the end of the study (Table 2). There was no significant difference in the 12‐month discontinuation rates between the two groups (12% in the self‐administration group; 22% in the healthcare provider group). Eighty percent of the self‐injections were given on the scheduled date, and none were given outside the appropriate interval. There was no explicit mention of failure of the method or severe adverse events. The study reported on injection problems experienced by the self‐administration group: 9% reported skin changes at the injection site; 6% had an acute reaction at the injection site; and 20% noted problems with the injection system (needle detachment from the syringe or difficulty in passing the medication through the needle because of resistance). There was no significant difference in the satisfaction rates towards their injectable method between the two groups. In addition, there was no significant difference in the proportion of women in either group who wished to continue the injectable method they had used during the study.

The third study by Stanwood et al.^15^ recruited women aged 18–40 years to a prospective cohort crossover study designed to compare the self‐administration of a monthly combined hormonal contraceptive intramuscular injectable at home with the administration of the same injectable by a nurse in a clinic setting. The study was designed for all participants to sequentially complete a teaching, home, and clinic phase. Both new users (*n* = 12) and women interested in continuing the method (*n* = 4) completed enrolment and an initial teaching visit. During the teaching visit, a nurse taught women to self‐inject intramuscularly in the anterior thigh and reviewed anatomical landmarks, proper sterile technique, and needle disposal; then, the nurse directly supervised and coached the women to ensure a successful first injection during the same encounter. Following the initial teaching visit, two women were lost to follow‐up and three women discontinued the study to use other contraceptive methods. Eleven women returned to the clinic 28 days later to assess proficiency with independent self‐injection when observed by the nurse, and were then provided with supplies sufficient for the next 3‐monthly injections to be completed by the women themselves at home. During the home phase, women documented the date of injection, and pain and anxiety they experienced at the time of injection, in a diary. A nurse contacted each participant on day 30 of each monthly cycle to confirm that they had self‐injected. After completing three injection cycles at home, women were requested to return for administration of the next three injections by a nurse. At the end of this clinic phase, women completed a survey to capture satisfaction and acceptability of administration in the clinic. Ten women (62.5%) completed all study procedures; it is unclear what proportion were new or continuing users at baseline.

The continuation rates were similar in both phases. All women who started the home phase (*n* = 11) completed all three injections as scheduled with no missed or late injections, although all participants received a telephone call reminder from the nurse; similarly, there were no reported problems with timely injection during the clinic phase (*n* = 10). There was no explicit mention of method failure or the experience of serious adverse events. Women reported similar experiences of low levels of pain and worry with self‐administration and nurse administration. They also reported equal satisfaction with the injectable during both the home and clinic phases. After the home phase, all women preferred self‐injection at home to nurse administration. At the completion of all study procedures, eight of the ten women noted a preference for self‐injection.

The overall certainty of the evidence as assessed by GRADE was low or very low. The study design contributed to the serious risk of bias for two studies (Table 1). Low‐certainty evidence shows that there was little or no difference in continuation rates when women self‐administered contraceptive injections in the RCT.^13^ For the two non‐RCT trials, the effect of the intervention on the continuation rate was uncertain because the certainty of the evidence was assessed as very low.^14^, ^15^ Safety was not estimable as there was no direct evidence identified for serious adverse events or other complications. For overall satisfaction towards the service and method, the effect of the intervention was uncertain as the certainty of the evidence was assessed as very low (Tables 3 and S1).

**Table 3:** Simplified summary of findings

What happens?	Healthcare providers providing contraceptive injections/implants	Women self‐administrating contraceptive injections/implants	Certainty of the evidence
**Continuation rates/re‐injection at 12 months (RCT)** There may be little or no difference in continuation rates when women self‐administer contraceptive injections/implants; however, the 95% CI shows both higher and lower continuation rates	304 per 1000	326 per 1000 (192–554 per 1000)^a^	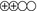 Low
**Continuation rates/re‐injection at 12 months (non‐RCT)** We are uncertain of the effect of the intervention on this outcome as the certainty of the evidence has been assessed as very low			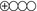 Very low
**Continuation rates/re‐injection at 3 months (non‐RCT)** We are uncertain of the effect of the intervention on this outcome as the certainty of the evidence has been assessed as very low			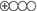 Very low
**Safety: serious adverse events** No direct evidence identified	Not reported	Not reported	
**Safety: other complications** No direct evidence estimable	Not estimable	Not estimable	
**Overall satisfaction with contraceptive service/method** We are uncertain of the effect of the intervention on this outcome as the certainty of the evidence has been assessed as very low			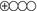 Very low

a 95% confidence interval.

## Discussion

### Main findings

We identified studies evaluating the self‐administration of either DMPA at 3‐month intervals, or a combined hormonal contraceptive injectable repeated once monthly, compared with administration by healthcare providers. It is important to note that the focus of this review was on the self‐administration aspect and not a comparison of the different modes of injection delivery. Results from one RCT demonstrated that there may be little or no difference in continuation rates between self‐administration and administration by healthcare provider; few conclusions about the effects on continuation can be drawn from the observational studies we included.

In all three studies, no serious adverse events, including method failures, were reported. None of the included studies were powered to detect differences in these otherwise rare events associated with contraceptive injectable use. Finally, it is notable that women's satisfaction with self‐administration was generally high and similar to healthcare provider administration across studies, but the certainty of this evidence was also assessed as being very low.

### Strengths and limitations

This is the first systematic review evaluating the self‐administration of contraceptive injectables. Our detailed analysis highlights critical knowledge gaps for the potential offer of self‐administered contraceptive injectables in the future.

The included studies had several limitations. In two of the three study designs participants were existing DMPA users, and were thus more likely to be motivated and were not representative of the larger population of DMPA users. Only three studies met the inclusion criteria, and all were small and conducted in high‐resource settings. These factors limit the generalisability of the results. There were also high dropout rates over time and none of the studies assessed follow‐up beyond 12 months. Furthermore, two of the three included studies were non‐RCTs, including one with a crossover design that followed the same set of women.

### Interpretation

Self‐administration appears to be an effective and safe alternative to health provider administration of injectable contraceptives; however, several considerations should be taken into account for future research and implementation.

Our main outcome of continuation rates was similar in both groups. The certainty of the current evidence is low, however, and in order to determine whether the effect is generalisable and reproducible we need additional well‐designed studies. Also, the included studies incorporated several mechanisms to remind women about the injection schedule, either through telephone reminders or written instructions. It is not possible to say how continuation rates would have been affected in the absence of these. Research to understand the need and most effective complementary strategies for well‐timed repeat self‐administration of contraceptive injectables is necessary. In addition, these strategies may vary according to the dosing schedule for the method (1 month versus 3 months) and the baseline experience of the woman with contraceptive injectables.

The observed outcome of similar continuation rates between the two groups prompts speculation on factors that would improve continuation with self‐administration. Context may be a factor. In low‐resource settings where ready access to healthcare providers in clinics may be a greater challenge, the provision of sufficient supplies and instructions for self‐administration may have a much more significant impact on the users’ continuation. Choice can be another factor. Future studies should consider a study design where women can choose self‐administration or provider administration, which may potentially produce greater continuation rates. Further exploration of various factors that drive women's decision‐making and contraceptive management could provide valuable information in assessing women's views towards self‐administration.

Method failure resulting in pregnancy is also an important outcome of effectiveness. None of the studies explicitly reported pregnancies amongst the contraceptive users, and no study followed women beyond 12 months. This may be because of the rarity of method failure, but future studies should consider reporting this outcome by increasing the sample size and follow‐up time to capture these potential events.

The fact that there was no reporting of severe adverse events may be a reflection of the overall safety of this method.^17^ The safety of self‐administration should also include the issue of syringe/needle disposal. It was noted in two of the included studies that the women in the self‐administration group were given supplies that included needle disposal containers. Problems with needle safety and disposal were not explicitly stated, but this should be another factor taken into account in future studies. It is a matter of not only personal safety but also public health safety, as improper needle handling and disposal can expose others to blood‐borne pathogens, particularly in regions of high HIV prevalence.

The satisfaction surveys in the included studies reflect that women are willing and able to self‐administer an injectable contraceptive; however, these included studies took place in high‐resource settings. A literature review examined the feasibility of self‐injection with DMPA‐SC in low‐resource settings.^8^ The review described the Uniject^™^ system that consists of a pre‐filled, non‐reusable, blister injection system with a bubble reservoir and an integrated ultrathin needle.^18^ This all‐in‐one injectable allows for easy use with minimal training, thus potentially simplifying the self‐injection process further. Implementation considerations include injection storage and waste disposal, and ensuring stakeholder support and a proper infrastructure that can facilitate the delivery of the injectable. It also noted the importance of initial as well as continued follow‐up of training to optimise the woman's ability to manage the self‐injection schedule.^8^ With the advent of a simplified delivery system, it is important to engage in research that compares this system with traditional delivery modes to further evaluate compliance, safety, continuation, and satisfaction towards self‐administration.

Although not a primary outcome, the studies did report additional benefits associated with self‐administration regarding convenience and potential cost savings for the woman, as well as for the health system. One of these studies compared the time and money spent on seeking/obtaining their DMPA injection (contraceptive behaviour) during the self‐administration phase versus the healthcare provider phase. All self‐administering women spent less than 30 minutes on contraceptive behaviour, whereas half of the women spent more than 30 minutes on contraceptive behaviour with the healthcare provider. The same study also noted that the subjects spent $10 more on contraceptive behaviour during the healthcare setting phase than the home phase (as a result of travel costs, time away from work, and childcare).^15^ With advanced supplies being given in all of these studies, it warrants future studies to take a closer look at the cost‐effectiveness aspect of self‐administration.

## Conclusion

For women self‐administering the contraceptive injection, the best available evidence shows that there may be little or no difference in continuation rates compared with administration performed by a healthcare provider. Although the absence of any serious adverse events precluded definitive conclusions on safety, it is well established that the contraceptive injectable is safe and complications are rare amongst users. Findings suggest that with appropriate information and training the provision of contraceptive injectables for women to self‐administer at home can be an option. Future studies should focus on larger sample sizes, engaging larger numbers of contraceptive users (non‐DMPA users, as well as new and old DMPA users) with longer follow‐up times, and should include low‐resource settings. Additional reporting of method failure alongside continuation rates, cost‐effectiveness of the self‐administration approach, and engagement of special populations, such as adolescents, should also be factored into future studies. The role of the Uniject^™^ delivery system in improving self‐administration outcomes also needs to be studied. This will increase the certainty of our initial findings that the self‐injectable contraceptive is a promising approach to make family planning methods accessible to potential users.

### Disclosure of interests

None declared. Completed disclosure of interests form available to view online as supporting information. The authors alone are responsible for the views expressed in this article and they do not necessarily represent the views, decisions or policies of the institutions with which they are affiliated. The mention of specific companies or of certain manufacturers’ products does not imply that they are endorsed or recommended by the World Health Organization in preference to others of a similar nature that are not mentioned. Errors and omissions excepted, the names of proprietary products are distinguished by initial capital letters.

### Contribution to authorship

All of the authors participated in the formulation of the methodology for this review. CK performed the literature search and reviewed all abstracts and full‐text articles. MSF carried out the analysis and critical appraisal of evidence. CK wrote the first draft of the article, and BG and MSF assisted in the editing and writing of the article.

### Details of ethics approval

This is a systematic review and does not require ethical approval.

### Funding

None.

## Supporting information


**Table S1.** GRADE table

**Appendix S1.** Search strategy.
