# Supplementary material for: Self‐administration of injectable contraceptives: a systematic review
Source: BJOG. 2016 Aug 23;124(2):200–8. doi: 10.1111/1471-0528.14248 (PMC5214286; doi:10.1111/1471-0528.14248)
Supplement: Supplementary file 1 — Table S1. GRADE table [file BJO-124-200-s001.pdf]

Table S1. GRADE table

Quality assessment							№ of patients		Effect			
**№ of studies**	**Study design**	**Risk of bias**	**Inconsistency**	**Indirectness**	**Imprecision**	**Other considerations**	**Women self-admin**	**Healthcare providers admin**	**Relative (95% CI)**	**Absolute (95% CI)**	**Certainty of the evidence**	**Importance**
continuation (non-interrupted use at 12 months) RCT												
1	randomised trial	not serious	not serious	not serious	very serious [2]	none	28/86 (32.6%)	14/46 (30.4%)	**RR 1.07** (0.63 to 1.82)	21 more per 1000 (from 113 fewer to 250 more)	⊕⊕◯◯ LOW	
continuation (non-interrupted use at 12 months) non-RCT												
1	observational study	serious [1]	not serious	not serious	very serious [2]	none	51/58 (87.9%)	50/64 (78.1%)	**RR 1.13** (0.96 to 1.32)	102 more per 1000 (from 31 fewer to 250 more)	⊕◯◯◯ VERY LOW	
continuation (non-interrupted use at 3 months) non-RCT												
1	observational study	serious [1]	not serious	not serious	very serious [2]	none	10/10 (100.0%)	10/10 (100.0%)	**RR 1** (0.83 to 1.2)	0 fewer per 1000 (from 170 fewer to 200 more)	⊕◯◯◯ VERY LOW	
Safety: serious adverse events												
0												
Safety: other complications												
1									not estimable	not estimable		
Satisfaction with the contraception method (want to continue this method) at 12 months												
1	observational study	serious [1]	not serious	not serious	very serious [2]	none	55/61 (90.2%)	50/55 (90.9%)	**RR 0.99** (0.88 to 1.12)	9 fewer per 1000 (from 109 more to 109 fewer)	⊕◯◯◯ VERY LOW	
Satisfaction with the contraception method (prefer to continue the method) at 3 months												
1	observational study	serious [1]	not serious	not serious	very serious [2]	none	10/10 (100.0%)	8/10 (80.0%)	**RR 1.24** (0.87 to 1.75)	192 more per 1000 (from 600 more to 104 fewer)	⊕◯◯◯ VERY LOW	
Satisfaction with the contraception method (recommend to a friend) at 12 months												
1	observational study	serious [1]	not serious	not serious	very serious [2]	none	57/61 (93.4%)	53/53 (100.0%)	**RR 0.94** (0.87 to 1.01)	60 fewer per 1000 (from 130 fewer to 10 more)	⊕◯◯◯ VERY LOW	
Satisfaction with the contraception method (recommend to a friend) at 3 months												
1	observational study	serious [1]	not serious	not serious	very serious [2]	none	9/10 (90.0%)	8/10 (80.0%)	**RR 1.13** (0.78 to 1.63)	104 more per 1000 (from 176 fewer to 504 more)	⊕◯◯◯ VERY LOW	
Satisfaction in location (at 3 months)												
1	observational study	serious [1]	not serious	not serious	very serious [2]	none	9/10 (90.0%)	9/10 (90.0%)	**RR 1** (0.75 to 1.34)	0 fewer per 1000 (from 225 fewer to 306 more)	⊕◯◯◯ VERY LOW	

MD – mean difference, RR – relative risk

1. High risk of bias in included study
2. One study only with few participants

High certainty evidence: Further research is very unlikely to change our certainty of the estimate of effect. **Moderate certainty evidence**: Further research is likely to have an important impact on the certainty of the estimate of effect and may change the estimate. **Low certainty evidence**: Further research is very likely to have an important impact on the certainty of the estimate of effect and is likely to change the estimate or any estimate of effect is very uncertain. **Very low certainty evidence:** Any estimate of effect is very uncertain
