# Supplementary material for: Self‐administration of injectable contraceptives: a systematic review
Source: BJOG. 2016 Aug 23;124(2):200–8. doi: 10.1111/1471-0528.14248 (PMC5214286; doi:10.1111/1471-0528.14248)
Supplement: Supplementary file 2 — Appendix S1. Search strategy. [file BJO-124-200-s002.pdf]

Appendix S1. Search strategy

Self and Contraception

PUBMED:

((((((((("injections"[MeSH] OR "injections"[tiab] OR "injectable"[tiab] OR "algestone acetophenide"[tiab] OR DMPA[tiab] OR deladroxate[tiab] OR "dihydroxyprogesterone acetophenide"[tiab] OR estradiol cypionate[tiab] OR estradiol 17 beta-cypionate[tiab] OR estradiol valerate[tiab] OR medroxyprogesterone acetate[tiab] OR medroxyprogesterone 17-acetate[tiab] OR 17-medroxyprogesterone acetate[tiab] OR mpa[tiab] OR NET-EN[tiab] OR NET-ENT[tiab] OR NET-OEN[tiab] OR norethindrone enanthate[tiab] OR norethindrone oenanthate[tiab] OR norethisterone enanthate[tiab] OR norethisterone oenanthate[tiab] OR ("medroxyprogesterone acetate"[MeSH Terms] OR ("medroxyprogesterone"[All Fields] AND "acetate"[All Fields]) OR "medroxyprogesterone acetate"[All Fields] OR "depoprovera"[All Fields]) OR ("medroxyprogesterone acetate"[MeSH Terms] OR ("medroxyprogesterone"[All Fields] AND "acetate"[All Fields]) OR "medroxyprogesterone acetate"[All Fields] OR ("depo"[All Fields] AND "provera"[All Fields]) OR "depo provera"[All Fields]) OR Curretab[tiab] OR Cycrin[tiab] OR "Depo-Provera"[tiab] OR "Depo Provera"[tiab] OR DepoProvera[tiab] OR Farlutal[tiab] OR Perlutex[tiab] OR Provera[tiab] OR Veramix[tiab] OR Clinovir[tiab] OR Gestapuran[tiab]) OR ("contraception behavior"[MeSH Terms] OR Contraception Behaviors[tw] OR Contraception Behaviour[tw] OR Contraception Behaviors[tw] OR (contraceptive behavior[tw] OR contraceptive behaviors[tw] OR contraceptive behaviour[tw] OR contraceptive behaviours[tw]) OR Contraceptive Usage[tw] OR Contraceptive Method Switching[tw])) OR ("contraceptive agents"[MeSH Terms] OR Contraceptive Agents[tw] OR contraceptives[tw])) OR ("contraception"[MeSH Terms] OR Contraception[tw] OR Contraceptive Methods[tw] OR Contraceptive Method[tw] OR Inhibition of Fertilization[tw] OR Fertilization Inhibition[tw] OR Birth Control[tw] OR Fertility Control[tw] OR Female Contraception[tw] OR male Contraception[tw] OR (contracept[tw] OR contracepted[tw] OR contraceptice[tw] OR contraceptices[tw] OR contraceptie[tw] OR contracepties[tw] OR contraceptifs[tw] OR contraceptiion[tw] OR contracepting[tw] OR contraceptio[tw] OR contraception[tw] OR contraception'[tw] OR contraception''[tw] OR contraception's[tw] OR contraceptionadvice[tw] OR contraceptional[tw] OR contraceptionin[tw] OR contraceptions[tw] OR contraceptiv[tw] OR contraceptiva[tw] OR contraceptivae[tw] OR contraceptive[tw] OR contraceptive'[tw] OR contraceptive's[tw] OR contraceptived[tw] OR contraceptively[tw] OR contraceptives[tw] OR contraceptives'[tw] OR contraceptives7[tw] OR contraceptiveshas[tw] OR contraceptiveson[tw] OR contraceptivity[tw] OR contraceptor[tw] OR contraceptors[tw] OR contraceptors'[tw] OR contracepts[tw]) OR (anticoncept[tw] OR anticonception[tw] OR anticonceptional[tw] OR anticonceptionals[tw] OR anticonceptivas[tw] OR anticonceptive[tw] OR anticonceptives[tw] OR anticonceptivo[tw] OR anticonceptivos[tw] OR anticonceptivs[tw]))) OR ("drug implants"[MeSH Terms] OR Drug implants[tw] OR drug implant[tw] OR Drug Pellets[tw] OR "levonorgestrel"[MeSH Terms] OR "norethindrone"[MeSH Terms] OR contraceptive implants[tw] OR (("progestins"[Pharmacological Action] OR "progestins"[MeSH Terms] OR "progestins"[All Fields] OR "progestogen"[All Fields]) AND only[All Fields] AND ("contraceptive agents"[Pharmacological Action] OR "contraceptive agents"[MeSH Terms] OR ("contraceptive"[All Fields] AND "agents"[All Fields]) OR "contraceptive agents"[All Fields] OR "contraceptives"[All Fields])) OR contraceptive implant[tw] OR progestogen implants[tw] OR etonogestrel implants[tw] OR Implanon[tw] OR (subdermal contraceptive

implant[tw] OR subdermal contraceptive implants[tw]) OR Norplant[tw] OR Jadelle[tw] OR Sino-implant[tw] AND Nexplanon[tw] OR Norprogesterones[tw])) OR ("intrauterine devices"[MeSH Terms] OR Intrauterine Devices[tw] OR Intrauterine Device[tw] OR Contraceptive IUD[tw] OR (intrauterine contraceptive device[tw] OR intrauterine contraceptive devices[tw]) OR Unmedicated IUDs[tw] OR Unmedicated IUD[tw])) AND ((("Self Administration"[Mesh] OR "Self Care"[Mesh]) OR "Consumer Participation"[Mesh] OR "Self Assessment"[Mesh] OR "Patient Participation"[Mesh] OR "Telemedicine"[Mesh]) OR ("self treatment*"[All fields] OR "self administer*"[All fields] OR "patient management*"[All fields] OR "self care"[All fields] OR "self management"[All fields] OR "self monitor*"[All fields] OR "home"[All fields] OR "telemedicine"[All fields] OR "self screen"[All fields]))

EMBASE

#	Search terms	Results
**1**	'self care'/exp OR 'self medication'/exp OR 'self evaluation'/exp OR 'telemedicine'/exp OR 'patient participation'/exp OR self treatment*:ab,ti OR self administer*:ab,ti OR 'patient management':ab,ti OR 'self care':ab,ti OR self management:ab,ti OR self monitor*:ab,ti OR patient treat*:ab,ti OR 'home':ab,ti OR 'telemedicine':ab,ti OR 'self screen':ab,ti	1547188
**2**	intrauterine AND devices OR contraceptive AND iud OR contraceptive AND iuds OR intrauterine AND contraceptive AND device OR unmedicated AND iuds OR unmedicated AND iud	1617
**3**	drug AND implants OR drug AND implant OR contraceptive AND implants OR progesteogen AND only AND contraceptives OR contraceptive AND implant OR etonorgestrel AND implants OR implanon OR nexplanon OR subdermal AND contraceptive AND implant* OR norplant OR jadelle OR 'sino implant' OR norprogesterones	2415
**4**	injections OR injectable OR dmpa OR algestone AND acetophenide OR deladroxate OR medroxyprogesterone AND acetate OR 'net en' OR 'net ent' OR noresterat OR norethindrone AND enanthate OR depoprovera OR 'depo provera' OR cycrin OR farlutal OR perlutex	2765
**5**	#2 OR #3 OR #4	6381
**6**	#1 AND #5	390

POPLINE

self AND depo provera

CINAHL

Self OR home AND)intrauterine device OR implant OR injection

CENTRAL

(self OR home) AND (contraceptive injection OR depo provera)
